# Neighbours and relatives: accounting for spatial distribution when testing causal hypotheses in cultural evolution

**DOI:** 10.1017/ehs.2023.23

**Published:** 2023-09-04

**Authors:** Lindell Bromham, Keaghan J. Yaxley

**Affiliations:** Macroevolution and Macroecology, Research School of Biology, Australian National University, Canberra, ACT 2601, Australia

**Keywords:** Galton's problem, phylogenetic non-independence, spatial autocorrelation, parasites, cultural evolution, humidity, tone

## Abstract

Many important and interesting hypotheses about cultural evolution are evaluated using cross-cultural correlations: if knowing one particular feature of a culture (e.g. environmental conditions such as temperature, humidity or parasite load) allows you to predict other features (e.g. language features, religious beliefs, cuisine), it is often interpreted as indicating a causal link between the two (e.g. hotter climates carry greater disease risk, which encourages belief in supernatural forces and favours the use of antimicrobial ingredients in food preparation; dry climates make the production of distinct tones more difficult). However, testing such hypotheses from cross-cultural comparisons requires us to take proximity of cultures into account: nearby cultures share many aspects of their environment and are more likely to be similar in many culturally inherited traits. This can generate indirect associations between environment and culture which could be misinterpreted as signals of a direct causal link. Evaluating examples of cross-cultural correlations from the literature, we show that significant correlations interpreted as causal relationships can often be explained as a result of similarity between neighbouring cultures. We discuss some strategies for sorting the explanatory wheat from the co-varying chaff, distinguishing incidental correlations from causal relationships.

## Graphical abstract

**Figure:**
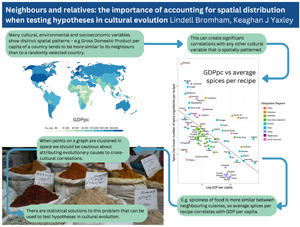

**Social media summary:** Causal claims are supported by correlations (e.g. parasites drive behaviour) but similarity between neighbours creates correlations (e.g. parasites correlate with Olympic medals, number of nurses, cheese consumption, traffic accidents).

## Introduction

Everyone is familiar with the adage ‘correlation does not imply causation’. A more nuanced version of that statement would be that correlation between two variables does not imply that one of the variables has a direct causal impact on the other; instead it often reveals an indirect link between them. Correlation analyses are often used to provide support for claims of causal relationships in cultural evolution (Claessens & Atkinson, 2022). Recent examples include: ecological threat influences motivation to punish norm-violators catalysing punitive religious beliefs (Jackson et al., 2021); larger speaker population sizes drive a reduction in the morphological complexity of languages (Kauhanen et al., 2023; Koplenig, 2019); women have stronger preference for men with more masculine faces under conditions of high offspring survival (Marcinkowska et al., 2019); and that moral vitalism (a belief in agentic spiritual forces of evil) is an adaptive cultural strategy that reduces rates of contagious disease (Bastian et al., 2019). While these studies do not rely wholly on the interpretation of statistical significance of cross-cultural correlations as their only form of argument, they all present significant correlations as providing valuable evidence for causal connections that supports a given hypothesis. Even where the language used conforms to formal structures of predictive causality (knowing the value of one variable allows you to predict the value of a second variable), the explanatory target of these studies is framed not simply in terms of revealing predictive associations (which may be indirect) but in inferring causality (the impact of one variable on the outcome of another).

Given that it is well understood that causality cannot be directly inferred from correlations, many such studies use a battery of approaches to interrogate the data (e.g. Jackson et al., 2021; Marcinkowska et al., 2019). Yet one major cause of ‘spurious’ correlations (i.e. statistically significant correlations that are not generated by a direct causal link between the tested variables) is either not addressed or inadequately countered: spatial autocorrelation of observations. Spatial autocorrelation is a critical factor in testing causal claims using cross-cultural data, because one of the common indirect links that causes ‘spurious’ correlations is shared environment. Neighbouring cultures tend to have similar environments, so any cultural variables that tend to be more similar between neighbours will also tend to correlate with environmental variables that are more similar between neighbours. Spatial autocorrelation – non-random distribution of trait values in space – can generate significant correlations between environmental and cultural variables, even when there is no direct causal connection between them. We can interpret such relationship in terms of indirect causal relationships – that is, some factor is causing the variables to covary, such as shared history or shared environments – but we may be led astray if we interpret the correlations as evidence for a direct impact of one of the variables on the other.

Recognising the influence of space on statistical inference is essential for developing and testing hypotheses about the evolution of human cultural diversity. More specifically, many important and interesting hypotheses about the influence of environment on cultural evolution are tested using cross-cultural correlations, for example the influence of humidity on the tonality of languages (Everett et al., 2015), the influence of parasite load on traditionalism (Tybur et al., 2016) and the influence of temperature on the spiciness of cuisine (Billing & Sherman, 1998). These hypotheses suggest that the evolution of particular cultural features is partly driven by the environment in which they arise. The focus here is on claims about environmental factors influencing the evolution of cultural diversity, rather than the influence of climate or environment on particular instances of human movement, population expansion or contraction, or events such as violent conflicts (Hsiang et al., 2013).

Testing hypotheses about environmental drivers of cultural diversity requires us to take the proximity of cultures into account. Nearby cultures share aspects of their environment and are also more likely to be similar in culturally inherited traits and shared history (Dow & Eff, 2008). This can generate indirect associations between environment and culture which could be misinterpreted as signal of a direct causal link, as we demonstrate in examples given below. People working in the field of cultural evolution are well aware of this problem: indeed the problem was clearly described over 130 years ago when Francis Galton suggested that to test a hypothesis about cultural evolution by comparing traits across cultures, ‘full information should be given as to the degree in which the customs of the tribes and races which are compared together are independent. It might be, that some of the tribes had derived them from a common source, so that they were duplicate copies of the same original’ (Galton, 1889a). While the eponym ‘Galton's problem’ is typically used to refer to the problem of phylogenetic non-independence (similarity between relatives), Galton also clearly identified spatial distribution as a potential source of statistical non-independence, and suggested that it would always be useful to look at the distribution of cultures on a map when evaluating evidence for hypotheses about cultural evolution (see also Loftin, 1972; Naroll, 1965). Yet, while the problem is widely recognised, spatial autocorrelation continues to complicate cross-cultural analyses, and compromises many statements of causality based on associations between culture and environment (Claessens & Atkinson, 2022).

## Congruent spatial patterns of diversity

The association between language diversity and biodiversity illustrates the problem of observations from nearby cultures being ‘duplicates’ rather than independent data points. Areas of high linguistic diversity tend to occur in areas of high biodiversity (Gorenflo et al., 2012; Sutherland, 2003). One explanation for this pattern is that the same environmental factors that support high species diversity also support high cultural diversity: tropical climates provide longer growing seasons allowing smaller, more localised cultural groups to persist, so more languages can be ‘packed’ into a given area (Hua et al., 2019; Nettle, 1996). It has been suggested that similarities in the spatial distribution between endangered species and endangered languages indicates that both biodiversity and cultural diversity are threatened by the same factors (Sutherland, 2003) and should be targeted by unified programmes to protect biocultural diversity (Loh & Harmon, 2005; Maffi, 2018). All things being equal, an area with more languages will also tend to have more endangered languages, particularly if areas of high language diversity support smaller speaker populations with smaller range sizes (Hua et al., 2019), since population size and area are associated with endangerment. When the spatial patterns of language endangerment and endangered species are taken into account, there is no significant association between the two (Bromham, 2023). Spatial autocorrelation may also underlie the reported correlation between language diversity and parasite diversity, which was interpreted as evidence that infection risk drives the divergence of cultures (Fincher & Thornhill, 2008). The diversity of parasites that cause human infectious diseases shows a latitudinal gradient (Dunn et al., 2010; Guernier et al., 2004), so it will tend to correlate with any other cultural variables that have a latitudinal gradient including language diversity (Bromham et al., 2018).

Spatial autocorrelation can generate significant correlations between cultures and environments at any scale of observation, whether global, regional or local. For example, New Guinea is a hotspot of both linguistic and biological diversity, but unlike the generally observed global pattern, there is a negative correlation between number of threatened languages and threatened mammals across the island (Turvey & Pettorelli, 2014). This correlation is driven (at least in part) by differences in history, environment and culture between the highlands and lowlands. Rates of language endangerment are highest in the lowlands of New Guinea, possibly owing to greater impact of colonisation or the influence of malaria, and lower in the highlands, where dense agriculture, lack of malaria and a more recent history of colonial impact may have promoted and protected language diversity. Yet there are more endangered mammals in the highlands, possibly because those areas support higher mammal diversity or because of human population pressure and hunting practices (Bromham, 2022b). Even if there is no causal connection between language endangerment and mammal species endangerment in New Guinea, because human cultures and mammal species are responding to separate threats in different areas, these distinct spatial patterns will generate a negative correlation between mammal endangerment and language endangerment through ‘duplicate’ observations: every time you sample a location from the lowlands, you will tend to find that it has high language endangerment and low mammal endangerment, and whenever you sample a location in the highlands it will have high mammal endangerment and low language endangerment, generating a negative correlation between language and mammal endangerment. When you take spatial proximity of observations into account, there is no significant association between mammal endangerment and language endangerment in New Guinea (Cardillo et al., 2015). In other words, knowing the level of mammal endangerment does not give you additional predictive power on language endangerment for an area, beyond knowing the location of the area and the values of language endangerment of its neighbours.

Spatial autocorrelation in cultural data could be interpreted in terms of ‘omitted variables bias’: if we fail to include information on the spatial distribution of our observations, then we are omitting an important cause of variation in our data, and this may lead us to think that other variables are causally related when they are only connected through the missing variable (sometimes referred to as a ‘back-door path’: Bulbulia et al., 2021; Pearl, 1993). However, the problem of spatial autocorrelation is a special case for two reasons. Firstly, it is pervasive: spatial autocorrelation affects virtually all cultural datasets. So, unlike canonical examples (e.g. Pearl & Mackenzie, 2018), spatial autocorrelation will tend to add ‘back-door paths’ that connect every cultural and environmental variable included in the analysis. Secondly, the problem of spatial autocorrelation is not simply that a key explanatory factor has been omitted: more seriously, it violates a common fundamental assumption of statistical tests, the independence of data points. Unlike an omitted variable, simply adding spatial information as a factor in the analysis (e.g. region, latitude) is not guaranteed to fix this.

Is the presence of autocorrelation among data points an issue of statistical inference, that demands technical solutions in the way we analyse our data, or an issue of causal inference, that requires a re-evaluation of the way we interpret the patterns in our data? There has been a long and vigorous debate about the relationship between statistical inference of significant associations in the data and the ability to make causal claims based on these patterns (Greenland, 2017; Hubbard et al., 2019; Rubin, 1991). Some researchers describe causal inference as a separate stage of data analysis from descriptive and predictive statistical tests (e.g. Laubach et al., 2021), but others consider causal inference part of the practice of statistical analysis, not separate to it. For example, many statistical techniques are described using causal terminology, such as analysis of time series (e.g. Oravecz & Vandekerckhove, 2023; Yang et al., 2018) or randomised control trials (e.g. Rubin, 1991; Tchetgen & VanderWeele, 2012). A recent report on data analysis from the US National Academies Research Council considers ‘Causal inference from observational data’ to be one of seven ‘inferential giants’ of data analysis, listed alongside the assessment of sampling biases, inference about tails and the reproducibility of analyses, suggesting that no fundamental distinction of type is made between the parts of the analysis that relate to statistical assessment of associations and the inference of causal relationships from those patterns (National Research Council, 2013).

In practice, many scientists do not draw a clear distinction between statistical inference and causal inference. Indeed, the motivation for conducting correlation analyses typically appears to be not simply to report patterns in the data but to uncover causal relationships between cultural traits, or between culture and environment. Because these tests function as both assessments of non-random patterns and support for causal hypotheses, they must be evaluated both from a technical statistical point of view (are the assumptions of the tests met by these data?) and from the viewpoint of causal inference (what is the nature of the association between variables?). For cross-cultural correlations, the assumptions of the tests are frequently violated by the non-independence of data points, and this impacts on the causal claims made on the basis of these analyses. The studies which are the focus of this paper – those that use cross-cultural correlations to test hypotheses about the causes of cultural diversity – are not solely aimed at description of statistical patterns in the data (e.g. association between the parasite load reported for different countries and aspects of their culture), but in explaining those patterns in terms of causes (for example, that the parasite load causes particular kinds of behaviours to evolve that reduce infection risk). Statistical tests are used to extract meaning from data: while they may reveal associations rather than causes, the results are routinely interpreted in terms of their support for hypotheses about causes (Pearl & Mackenzie, 2018; Shipley, 2016). Indeed, significant statistical test results (typically *p* < 0.05) are generally taken as an essential step in supporting causal claims. Therefore the application and interpretation of statistical tests are at the core of inferring causality from cross-cultural data.

## Inferring causal connections from cross-cultural correlations

Correlation analysis was invented to detect causal relationships. In the paper that introduced the procedure, using the example of correlation between the size of structures on the same individual, Galton said: ‘It is easy to see that co-relation must be the consequence of the variations of the two organs being partly due to common causes. If they were wholly due to common causes, the co-relation would be perfect, as is approximately the case with the symmetrically disposed parts of the body. If they were in no respect due to common causes, the co-relation would be nil. Between these two extremes are an endless number of intermediate cases’ (Galton, 1889b). Correlation owing to ‘common causes’ can include indirect causal relationships, where the values of different variables follow predictable relationships owing to their covariation with another (hidden) variable.

Significant correlations between environment and culture are easy to find, yet the nature of the causal connection between correlated variables is not always obvious. For example linguistic diversity is significantly positively correlated with fatal traffic accidents (Roberts & Winters, 2013), bird diversity is significantly correlated with religiosity (Bromham et al., 2018), rainfall with individualism (Davis, 2012) and global CO_2_ with homicides (Munshi, 2018). So many cultural traits show congruent spatial patterns that it is not difficult to find significant correlations in cross-cultural datasets (Calude & Longo, 2017). Naturally, a given study will include variables of interest, but will not exhaustively check other variables have equivalent or greater predictive power. For example, a significant correlation between the frequency of beards and parasite load across 25 countries has been interpreted as evidence for a causal impact of environment on culture, such that beards may function in mate choice as markers of relative health (Dixson & Lee, 2020; Pazhoohi & Kingstone, 2020). Beard frequency shows an even stronger correlation with variables that were not included in the study, including population size, the number of nurses and midwives per 1000 people, belief in the devil and per capita alcohol consumption (see Supplementary Information for details). Variable choice is subjective but shapes interpretation of causal connections from significant correlations.

All correlations are caused by something, but not all (perhaps relatively few) reveal direct causal relationships between the correlated variables. A decision to accept the correlation between beards and parasites as evidence for a direct causal connection but not to interpret the association between beards and nurses as indicating a causal relationship is not based on any difference in the quality of evidence or the strength of the statistical test. It could be argued that we should preferentially accept statistical evidence if it relates to a pre-existing hypothesis that predicts a particular association or if there is a plausible explanation why such a correlation should exist. Nonetheless, we do not have any statistical basis on which to interpret some significant correlations as indicating a causal relationship (e.g. beards and parasites) while rejecting similarly significant correlations as spurious (e.g. beards and nurses). Any such judgement on causality is not directly connected to the statistical test itself, but is a statement of prior belief in the plausibility of particular relationships. In that case, it is the prior belief rather than the statistical test *per se* that is being used to support one causal explanation over the other. Yet statistical tests are widely considered to be essential tools for evaluating causal claims. Papers that use cross-cultural correlations to test hypotheses about the causes of cultural diversity are aimed not solely at description of statistical patterns in the data, but at explaining those patterns in terms of causes (for example a high environmental parasite load causes the evolution of behaviours that reduce infection risk, or favour the acquisition of traits that indicate relatively low individual parasite load).

There are three intertwined problems in using cross-cultural correlations to test hypotheses about the causes of cultural differences. Firstly, neighbouring cultures tend to share many traits and this spatially clustered distribution will generate significant correlations even in the absence of any direct causal connections. For example, there is a significant correlation between the number of Nobel prizes awarded per country and the number of IKEA stores. It is unlikely that anyone would put forward a direct causal explanation for this relationship, but they would instead explain this pattern in terms of spatial autocorrelation: both Nobel prizes and IKEA stores are not randomly distributed across the globe, but are historically biased towards northern Europe. Including many northern European countries in the analysis, each of which has many Nobel prizes and many IKEA stores, generates the impression of a link between Nobel success and flat-packed furniture (Maurage et al., 2013). Secondly, related cultures will be more similar in many, if not most, variables so failing to take phylogenetic non-independence into account can also generate correlations between variables that have no direct causal connection. In practice, it is often difficult to clearly separate out patterns of similarity owing to proximity from patterns of similarity owing to shared ancestry, since neighbours are often also relatives. In the example just given, Scandinavian countries share many aspects of their environment (low average temperature, low parasite load), but also share a cultural heritage (which influences culture and commerce, e.g. lots of IKEA stores, high chocolate consumption). Thirdly, many cultural traits and environmental traits covary, so connection between any of these traits will generate many other significant correlations (e.g. Nobel prizes are significantly correlated to chocolate consumption; Messerli, 2012). Owing to similarities between neighbouring and related cultures, these problems are likely to be so common in cross-cultural analyses that its safest to assume that they are essentially universal. Furthermore, because these factors interact, it may be difficult to easily partition out the effects of phylogenetic non-independence (relatedness), spatial autocorrelation (proximity) and co-variation.

In this paper, we focus on spatial autocorrelation, as it has received relatively less attention in the cultural evolution literature than phylogenetic non-independence (e.g. Bromham, 2022b; Evans et al., 2021; Mace & Holden, 2005; Mesoudi, 2016) and covariation (e.g. Bulbulia et al., 2021; Deffner et al., 2022). Luckily, there are statistical techniques that can deal with all three problems, helping us sort the explanatory wheat from the covarying chaff.

## Spatial autocorrelation in cross-cultural analyses

Any variables that have spatially clustered values can lead to indirect correlations that could be misinterpreted as a sign of a causal relationship. If variables are plotted on a graph and the data points cluster by region, or if the values of the variables are clustered when plotted on a map, then the data points are spatially autocorrelated and do not satisfy the basic assumption of statistical independence. Any *p*-value from a correlation where the data points are clustered by location is meaningless, because the assumptions of the test have been violated. As a simple illustration of the impact of non-random spatial distribution on statistical tests, we can show that languages that use a single semantic category for hand and finger (Brown, 2013) are significantly more likely to be endangered or no longer spoken than languages that have separate words for hand and finger (see Supplementary Information). Why? It is difficult to imagine that anyone would seriously invoke a causal connection between having a single category for hand–finger and language loss, despite the significant *p*-value. If we plot languages on a map, the cause of the strong correlation is clear: most of the languages that are recorded as having a single category for hand–finger are from North America and Australia, two continents that have suffered particularly high rates of language loss owing to brutal colonial suppression of Indigenous languages (Figure S4). However, if we fit a model which takes this spatial autocorrelation into account, then the apparent relationship between language endangerment and having a single category for hand and finger is no longer significant (see Supplementary Information).

Spatial distribution should always be considered in evaluating hypotheses about human cultural evolution. For example, the observed correlation between tonality of languages and relative humidity has been interpreted in terms of coevolution of human physiology, language and environment, based on the observation that drier air presents challenges for the generation of precise differences in tone (Everett et al., 2015). This is a critical case study with important implications for understanding the evolution of human language, because it suggests that features of language can be shaped by environmental variation (Everett et al., 2016a). More broadly, it is a key example of an adaptive hypothesis in human cultural evolution (Lupyan & Dale, 2016).

**Figure 1.**
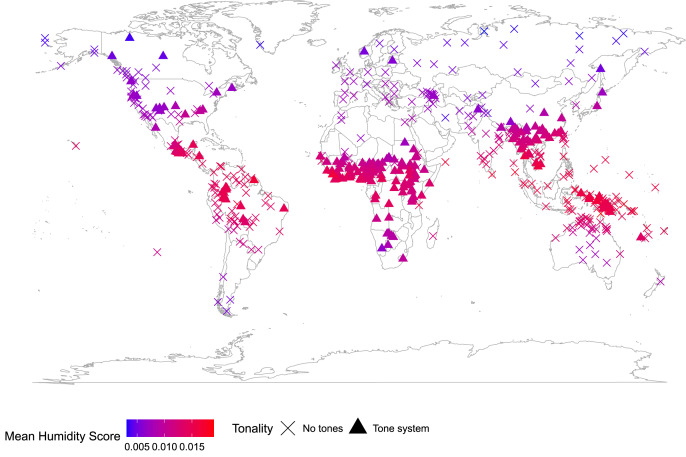
Global distribution of tonal languages. Language data from the World Atlas of Linguistic Structure (WALS) database for the 527 languages with information on this variable (13A) (Maddieson, 2013). A triangle marks the geographic point associated with a language recorded as having tonal features (220 languages), and a cross marks the geographic point associated with a language recorded as having no tonal features (307 languages). The colour of the point represents the predicted mean humidity score at that point. Logistic regression *N* = 527, *β* = 0.301, 95%CI [0.123–0.483], OR = 1.351, 95%CI [1.131–1.62], *z* = 3.29, *p* = 0.001, d.f. = 525, AIC = 708.99. See Supplementary Information for details of data analysed. Map from South (2017).

Plotting tonal languages on a map shows that the majority are distributed in areas with relative high humidity (Figure 1). This is not surprising, for two reasons. Firstly, language diversity shows a latitudinal gradient, so there are more languages in tropical areas. Even if we sample languages by putting all of the world's languages in a hat and drawing them out at random, we would expect that more of the languages we sample will be closer to the tropics, and therefore be in areas of relatively higher humidity. Secondly, tonal languages tend to be related to other tonal languages, and related languages tend to cluster in space (Collins, 2016). Any feature that is found in multiple members of some language families, but is absent in other language families, will probably cluster in space, and therefore also potentially correlate with spatial patterns of environmental variation. Tonal languages predominately cluster in sub-Saharan Africa, East Asia and South East Asia, areas that also have relatively high humidity. Therefore, as the study's authors note, the data should be analysed accounting for both phylogenetic relationships and spatial distributions (Everett et al., 2016b).

There is a significant correlation between tonality and humidity (*N* = 527, *z* = 3.29, *p* = 0.001; see Supplementary Information for details of data and analysis). However, the residuals of this logistic regression are significantly associated with distance (Moran's *I*; *N* = 527, observed = 0.152, expected = −0.002, SD = 0.007, *p* < 0.001), indicating spatial autocorrelation in the data (for an explanation of Moran's *I*, see below). Is spatial autocorrelation alone enough to explain the higher representation of tonal languages in areas of high humidity? Fitting a logistic regression model with tonality as the outcome variable, mean humidity as a predictor variable and a structured variance–covariance matrix constructed from the great circle distance between the languages with an exponential decay process suggests that having information on humidity does not have any predictive power for tonality once the spatial distribution of languages is taken into account.

It is important to emphasise that showing that the correlation between humidity and tone is not significant once spatial autocorrelation is taken into account does not disprove the general hypothesis that language may evolve in response to environment, nor the specific hypothesis that tonal languages are more likely to arise in humid areas. These explanations might be plausibly supported by other lines of evidence (Ladd, 2016). However, it does tell us that these cross-cultural data do not provide convincing support for accepting the hypothesis that humid air promotes the evolution of tone in language, because we can explain the observations without invoking any causal connection between tone and humidity. An alternative explanation – that a biased spatial distribution of related languages can account for the co-occurrence of humidity and tone – provides an equally good explanation of the data, so the data give us no cause to prefer an explanation involving humidity as a cause of tonality.

Because related languages cluster in space, correlations between language features and environment are not surprising; in fact they are very common. For example, employing logistic regression we get as strong a *p*-value for the correlation between humidity and passive voice (*p* < 0.001) as between humidity and tonality (*p* = 0.001). Languages in areas of low humidity are also significantly more likely to have a past tense (*p* = 0.017; see Supplementary Information). Similarly, tonal languages will correlate with many other features of the environment, for example with amphibian species richness (*p* = 0.003), but this is unlikely to indicate a direct causal impact of frogs and salamanders on language tonality (or vice versa). The challenge with using cross-cultural data to infer causal links between environment and human cultural variables is that that we could continue to add variables that show the same spatial distribution as humidity, tonal languages, passive voice and amphibians, and it would be difficult to tell from those analyses which (if any) of the included variables were driving the significant correlations. If our data has spatial patterning, but we do not know what the cause of that spatial patterning is, there is no limit to the number of potential ‘back-door paths’ we could add to our analysis (variables that form indirect links between our target variables).

Spatial autocorrelation will also complicate the search for links between genetic variants and language diversity. As humans spread over the landscape, they take their languages and their genes with them, and both accumulate changes over time and space. We should therefore expect many different genetic variants to correlate with language variants. Genes associated with brain function show correlation with tonal languages (Dediu, 2021; Dediu & Ladd, 2007). So do mitochondrial genetic variants (Collins, 2017). While a plausible case has been made for the association between the correlated brain genetic variants, mitochondrial genes (which are associated with basic cellular metabolism) seem unlikely to have any causal association with tonality. Instead, mitochondrial genes are common markers of population history which flow with the tide of people and cultures. Any test of the significance of association between genes and language must do so by comparison with the expected background level of covariation between genes, language and space, to discount the expected relationships between genes and language that come ‘for free’ from human population history (Barbieri et al., 2022; Dediu, 2021; Ladd et al., 2015).

## Untangling links between cultural and environmental variables

If we wish to identify causal relationships between aspects of environment and culture, it is not sufficient to identify variables that are significantly correlated. Many environmental and cultural variables are strongly colinear, following the same general trends as each other, making it very easy to find significant correlations in cross-cultural observations. We can illustrate the problem of covariation by considering a well-known example of an adaptive cultural evolution hypothesis: that spicy food is promoted by cultural selection in areas of high parasite load because spices have anti-microbial properties that reduce the risk of food-borne disease (Sherman & Billing, 1999). This hypothesis has been supported by correlation between average temperature and average spice use from samples of recipes from different countries, on the grounds that food spoilage is a greater problem in hotter climates, potentially increasing the benefit of adding anti-microbial spices (Billing & Sherman, 1998). That there is a relationship between average number of spices per recipe and temperature is not in doubt, but observing that relationship does not tell us that temperature itself has a direct causal role in driving patterns of spice use. In fact, over half the variation in spice use can be explained by distance between cuisines and by their relationship to other cuisines (Bromham et al., 2021). Once the association between spice, temperature and parasite load that comes from having neighbours and relatives with similar cuisines and similar environments is taken into account, there is no additional association between spice, temperature and parasite load. In other words, we have as much power to predict average spices per recipe based on information about neighbouring cuisines as by knowing the temperature or parasite load of the area.

**Figure 2.**
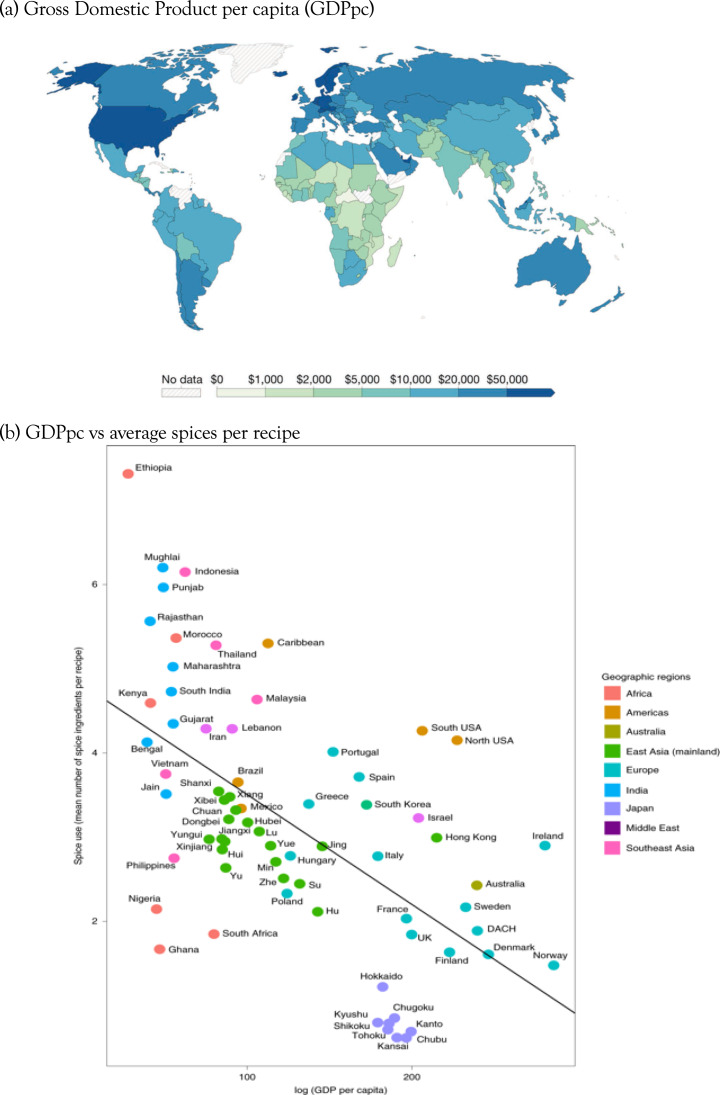
Spatial patterns lead to significant correlations between cultural variables. (a) Values of gross domestic product per capita (GDPpc) per country. Map from OurWorldInData.ora/economic-growth based on multiple sources compiled by World Bank, 2019 figures expressed in international-$ at 2017 prices, published under CCBY licence. (b) Average number of spices per recipe for national and sub-national regions plotted against GDPpc: reproduced from Bromham et al. (2021).

Including spatial data in the analysis allows us to winnow some variables that have no additional explanatory power compared with simply knowing the location of the observations, and focus on relationships that have addition predictive power. Average spices per recipe does correlate with reported rates of foodborne illness and childhood diarrhea, above and beyond the covariation owing to proximity and relatedness, which could be interpreted as supporting an association between spice and risk of foodborne infection. However, poor health outcomes of many kinds tend to covary together, and are strongly associated with relative wealth, which tends to be more similar between neighbouring countries (Figure 2). Gross domestic product per capita (GDPpc) is a stronger predictor of average spice use than foodborne illness, which means that any factors that are more similar between relatively poorer countries will also correlate with spice use, including a wide range of poor health outcomes (e.g. fatal traffic accidents are strongly correlated with average spice use; Bromham et al., 2021). Any feature of cultures that correlates with GDPpc is also likely to correlate with everything else that correlates with GDPpc, including pathogens, disease outcomes, life expectancy, education, environmental modification and population density (Bonds et al., 2012; Kummu & Varis, 2011; Smith et al., 2003). Because nearby cultures are more similar in many respects, including relative wealth, it is easy to find correlations between GDPpc and cultural variables. For example, GDPpc is significantly correlated with belief in the devil, cheese consumption and Olympic medals (see Supplementary Figure S3).

**Figure 3.**
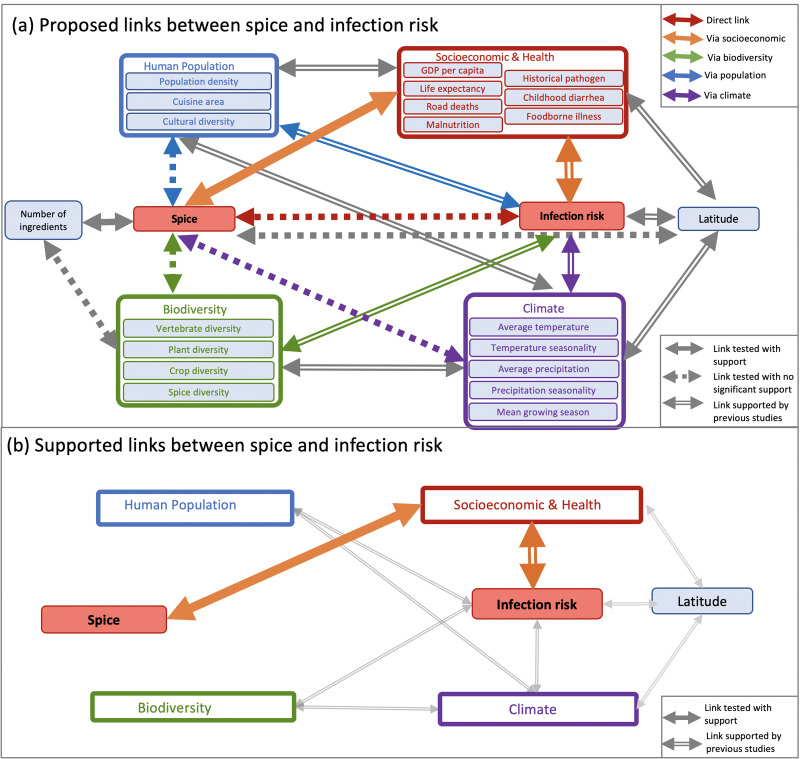
Potential links between variables can be represented graphically. In this example, the (a) proposed causal link between infection risk and spicy food (Sherman & Billing 1999) could also be explained by indirect paths through the covariation of population, diversity and climate, but (b) indirect paths via socioeconomic variables provide significantly stronger support than any other tested links between infection risk and spice. Redrawn from Bromham et al. (2021).

Drawing causal diagrams can help to explore proposed causal relationships between culture and environment (Figure 3), building complicated ‘horrendograms’ of links between culture, environment, populations and diversity based on cross-cultural correlations, prior knowledge or alternative hypotheses (Barbrook-Johnson & Penn, 2022). We can then compare the explanatory power of variables. In this example, we can conclude that the data give us no reason to attribute a causal link between spice and temperature or parasite load, because we can explain that correlation with proximity and relatedness: nearby and related cultures are more similar to each other in all three variables. We can also show that only the socioeconomic variables have significant explanatory power above and beyond their covariation with space, relationships and other cultural variables. Yet even after we have eliminated most variables, we are unable to answer the question ‘what *does* explain variation in spice use’, because there is no limit to the number of additional variables we could add that show congruent patterns. If we continue to add variables, we would eventually find one that had a stronger relationship with spice, above and beyond its covariation with relatedness, proximity and other socioeconomic, environmental and cultural variables.

Interrogating the tangled web of cross-cultural correlations does not tell us about the reasonableness of the underlying causal hypotheses (Roberts & Winters, 2013; Roberts et al., 2020), but it does allow us to evaluate whether a hypothesis is well supported by particular datasets. There is nothing inherently wrong with the hypothesis that human cultures have adapted their cuisines to respond to local environments, and that ingredients with antimicrobial properties might be favoured where risk of food-borne illness is high. And it may be that the analysis fails to capture the appropriate variable, for example the average number of spices per recipe does not capture the amount of potentially anti-microbial ingredients added to food (e.g. adding a lot of chilli might be more effective than adding a small amount of many other spices), or that the measures of food-borne illness do not target all forms of intestinal infection (Hagen et al., 2023). However, we can conclude that these cross-cultural data do not provide convincing support for a causal link between spice use and infection risk because the correlations between spice use, parasite load, temperature and foodborne disease can be explained simply in terms of nearby countries sharing many factors in common including cuisine, climate, wealth and health. An alternative approach to testing this hypothesis might be to make predictions about other potentially antimicrobial ingredients: for example we should also expect to see that cultures add other antimicrobial ingredients to their food, yet there is no association between parasite load and vinegar or alcohol (Bromham et al., 2021). Or we could extend the test to other dietary practices that could be interpreted as providing a level of protection against foodborne infection, for example forbidding high-risk foods; however, no correlation was found between food taboos from many cultures or religions and pathogen prevalence (Wormley & Cohen, 2022).

## Practical solutions for dealing with spatial autocorrelation

Spatial autocorrelation generates challenges for testing hypotheses in cultural evolution whenever the value of a variable is clustered by location, so that nearby cultures tend to be more similar to each other than they are to more distant cultures. It is not just a problem of global analyses, but can occur at any scale; for example, associations between language endangerment and species endangerment occur at both global and local scales, but both may be generated by spatial autocorrelation (Cardillo et al., 2015; Hua et al., 2019). Methods for addressing spatial autocorrelation have been long discussed in other fields, such as econometrics (e.g. Elhorst, 2010), ecology (e.g. Dale and Fortin, 2002) and geography (e.g. Getis, 2008). There are many published guides to addressing the general problems of covariation in cultural data when testing hypotheses in cultural evolution, for example books and online lectures by McElreath (2020), workflow suggestions by Bulbulia (2022) and a helpful online tutorial by Scott Claessens (https://scottclaessens.github.io/blog/2022/crossnational/), although these resources are not specific to dealing with spatial patterning in cultural variables. Alternatively, there are many useful guides to analysis of spatially autocorrelated data from other fields, particularly ecology (Dormann et al., 2007) and geography (Akbari et al., 2023). However, although the problem is widely acknowledged in the field of cultural evolution studies, as yet there has been a lack of consistent and effective methods for dealing with spatial autocorrelation applied in tests of cultural evolutionary hypotheses from cross-cultural comparisons (Claessens & Atkinson, 2022; Pollet et al., 2014).

A useful first step is to diagnose the problem by asking whether your data is spatially patterned. If you plot your variables on a map, do they cluster in space (Figures 1 & 2)? Are two nearby cultures likely to have more similar values for this variable than two cultures chosen at random? Another qualitative diagnostic is to plot all of the data and label them by culture, region or country (e.g Figures S1 and S2). Are the data points randomly scattered with respect to location or do they cluster by region? Are neighbouring countries more likely to occur in similar regions of the co-ordinate space? If data are not randomly distributed with respect to location, then this violates the assumption of any standard statistical test that the residuals should be randomly distributed. Examining residuals is another approach to diagnosing spatial autocorrelation in data: if a model is fitted to the data, but there is a clearly biased distribution of the residuals from that model, this suggests that the data violate assumptions of statistical independence. Drawing causal diagrams, where relationships between variables are represented by arrows (e.g. Figure 3), can be a useful guide to hypothesis testing, whether these are used informally to represent and clarify the relative support for proposed links (Bromham et al., 2021; Roberts & Winters, 2013; Roberts et al., 2020) or more formally, for example as the basis for Bayesian networks or path analysis (McElreath, 2020; Pearl & Mackenzie, 2018; Shipley, 2016). Directed Acyclic Graphs could be applied to modelling the component of variation owing to spatial distribution (Akbari et al., 2023), although as yet there are few, if any, examples of its application to accounting for spatial autocorrelation in cross-cultural data.

There are a number of more formal tests for spatial autocorrelation within a dataset. A Mantel test is often used to detect spatial patterning in data, by looking for a correlation between a matrix of pairwise differences between observations and a matric representing the pairwise spatial distances between those observations (e.g. Passmore & Jordan, 2020; Roberts et al., 2015; Saslis-Lagoudakis et al., 2014), although application of partial Mantel tests may not be a reliable solution to analysing spatially non-random data (Nunn et al., 2006). Moran's *I* can also be used to test whether the values of a set of spatially distributed observations are more dispersed or clustered than would be expected if the values were distributed randomly among the observations. A significant *p-*value and positive *z*-score suggest that the null hypothesis that data points are randomly distributed with respect to space can be rejected, because observations are more spatially clustered than would be expected by chance. If the *p-*value is significant but the *z*-score negative, then the observations are more spatially dispersed than expected. Moran's *I* can be calculated for individual variables but is also routinely applied to the residuals of multivariate linear and generalised linear models. The test can be easily implemented in R using packages such as _*DHARMa* (Hartig, 2022) and *ape* (Paradis & Schliep, 2019).

A common approach to dealing with spatial autocorrelation in cross-cultural data is to add spatial data to the analysis by assigning data points to regional groups or ‘bands’ and adding this as a factor in the analysis. An alternative approach has been to select sparse samples of spatially distributed cultures, for example sampling one per region or drawing from the Standard Cross-Ccultural Sample (SCCS). Yet neither of these approaches will solve the problem of spatial autocorrelation of variables if cultures within each group, band or region still show evidence of clustering by location (Bromham, 2022a; Eff, 2004; Loftin, 1972; Mace and Pagel, 1994; Pollet et al., 2014). We expect cultures within each SCCS region to be more similar to each other than to cultures in other regions; for example, Bau Fijians and Western Samoans are likely to have more similar values of many aspects of culture and environment than either does to Copper Eskimo and Aleut, and vice versa. Including any form of location information as a factor in an analysis (e.g. latitude and longitude) is effectively adding a ‘hidden variable’ to capture spatial patterns in the data, by standing in for some unknown spatially patterned variable that causes indirect links between environment and culture. This approach assumes that autocorrelation will decline linearly with distance, although extra terms – such as quadratic terms for location information – can be added to model non-linear relationships between outcome variables and space, but this will exacerbate model complexity. However, adding spatial information as covariates in a model does not mitigate the underlying assumption of the test that data points are independent so that that the residuals should not show any bias. Conley standard errors are increasingly being used as a mitigation of spatial autocorrelation in analyses of cross cultural data (e.g. Schulz et al., 2019); however, they may not have sufficient impact on reducing the false positive rate (Claessens & Atkinson, 2022) and do not address the related problem of phylogenetic non-independence.

One common approach is to implement simultaneously autoregressive models (SAR). SARs assume that the value of a variable at a particular site is in part influenced by the value of that same variable at neighbouring sites. By providing a spatial weights matrix – some measure of the connectivity between sites – SARs estimate the relative contribution of spatial autocorrelation to values of the outcome variable in a generalised least-square regression (see Kissling & Carl, 2008). One of the advantages of this approach is that spatial weights matrices can be produced using different covariance functions – models for how autocorrelation changes with distance – such as an exponential or Matérn process function. These missing models can then be compared using standard model selection techniques, like the Akaike information criterion to identify the covariance process which best explains the autocorrelation present in the data. SARs can also be implemented in path analysis, by being incorporated into the paths of structural equation models (Skeels et al., 2020), which may provide a useful tool for modelling phenomena where there are complex interdependences between predictor variables and potentially multiple outcome variables. By utilising variance/covariance matrices calculated across a range of lag distances, spatially explicit structural equation models can show how path coefficients change across different spatial scales (e.g. using the R package ‘sesem’; Lamb et al., 2014).

An analytical solution that deals with all three interrelated problems of relatedness, proximity and covariation has been successfully applied to cross-cultural analyses (Bromham et al., 2018, 2021, 2022; Hua et al., 2019; Skirgård et al., 2023). Phylospatial analysis that incorporates a covariance matrix for both spatial and phylogenetic distance between observations offers the opportunity to explore patterns in the data (Dinnage et al., 2020; Freckleton & Jetz, 2009; Hua et al., 2019). Such an analysis estimates the amount of signal owing to both space and relationships, and if there is no autocorrelation in the data then that covariation matrix will be set to zero and the analysis is equivalent to statistical independence between datasets. Estimating the covariance owing to proximity and relationships allows the data to speak: if patterns of variation do not correspond to proximity or relationships then the covariance matrices owing to phylogeny and distribution will not influence the analysis. It is not necessary to assume, *a priori*, either that cultures represent independent data points owing to separate instances of adaptation to shared environmental conditions (Thornhill & Fincher, 2013) or that observations are confounded by non-independence owing to descent or shared environment. The need to incorporate information on relatedness and proximity does not need to be settled by argument alone, but by testing for evidence of spatial autocorrelation (e.g. Dobson & Gelade, 2012) or phylogenetic signals in the data (e.g. Roberts et al., 2015). The degree to which variation is explained by spatial distribution or phylogenetic relationships can be an interesting outcome of such an analysis in its own right. Analysis packages that allow for linear, generalised linear and mixed models to be fitted with a variety of covariance functions can be used to model spatial autocorrelation (e.g. ‘*glmmTMB* ‘R package: Brooks et al., 2017). Alternatively, many published studies provide custom code for phylospatial analysis of cross-cultural data (e.g. Bromham et al., 2022; Hua et al., 2019; Skirgård et al., 2023).

There is no single analytical solution to analysing cross-cultural data to extract meaningful information about causal relationships (McElreath, 2020). However, failure to account for relationships and proximity may lead researchers down unhelpful explanatory paths. When the observations in an analysis come from entities that are related by descent (such as species, languages or cultures) then the relationships between observations will confound our ability to identify causal connections, whether we have a phylogeny or not (Felsenstein, 1985; Mace & Holden, 2005; Mace & Pagel, 1994). If cultures tend to be more similar to their relatives and neighbours than they are to randomly selected cultures, then failing to incorporate information on location relies on the implicit assumption that knowing about the state of a variable in nearby cultures gives you no predictive power for the state of their neighbours. Any form of spatial data is better than none, just as any information on relationships will be better than assuming there are no patterns of similarity by descent in the data. Information on similarity owing to descent does not necessarily require a resolved bifurcating tree (Bromham, 2022a); for example, it may be possible to use hierarchical language classification as a way of estimating covariation owing to relationships between cultures (Hua et al., 2019). Similarly, point locations for cultures or languages may not be a perfect representation for proximity, but they are a lot better than an implicit assumption that all cultures are equidistant. Accounting for spatial distribution does not by itself tell us about the veracity of any causal relationships among variables, but it can provide a plausible alternative explanation for the association between variables, by demonstrating that we could get the same relationship simply by neighbours being more similar to each other in many ways, even if there was no causal connection between the variables. Alternatively approaches using networks (Evans et al., 2021) or directed acyclic graphs to make explicit the potential causal connections between variables (Bulbulia, 2022; Deffner et al., 2022) may also help researchers to formulate hypotheses (Roberts et al., 2020) and find the signal of causal connections between culture and environment.

## Conclusions

Anyone who is interested in investigating possible environmental drivers of cultural variation cannot afford to ignore spatial autocorrelation, because it can create strong correlations between variables that have no direct causal connection. Incorporating spatial distribution in any tests of cultural evolution hypotheses is essential to avoid being led astray by indirect associations between variables. Showing that the relationship can be explained by phylogeny or proximity does not disprove a hypothesis. These relationships may well be pointing to important drivers of cultural evolution, but a cross-cultural correlation does not add meaningful support to causal hypothesis if it can be explained by the correlation that comes ‘for free’ from proximity and/or relatedness alone. Given the high degree of covariation between aspects of cultural diversity and environment, we have to work harder to prove causality above and beyond the explanation that neighbours tend to be similar in many aspects of their cultures, whether or not those factors are causally related.

## Data Availability

All data is from public or previously published sources (see Table S1) and is analysed using freely available software, as cited in the text. The tables of variables analysed and their sources are included in the Supplementary Information. Custom code and data required to repeat the analyses is available at: https://github.com/keaghanjames/relatives_and_neighbours/tree/main

## Supplementary material

For supplementary material accompanying this paper visit https://doi.org/10.1017/ehs.2023.23. click here to view supplementary material
