# Supplementary material for: Neighbours and relatives: accounting for spatial distribution when testing causal hypotheses in cultural evolution
Source: Evol Hum Sci. 2023 Sep 4;5:e27. doi: 10.1017/ehs.2023.23 (PMC10565196; doi:10.1017/ehs.2023.23)

Supplementary information for:

Neighbours and relatives: accounting for spatial distribution when testing hypotheses in cultural evolution

Lindell Bromham, Keaghan Yaxley

Macroevolution and Macroecology,

Research School of Biology,

Australian National University, Canberra ACT 2601 AUSTRALIA

Detail of analyses described in the main text:

1. Beards and parasites
2. Belief in the devil and parasites
3. Hand/finger distinction and endangerment level
4. Tonal languages and humidity

Code availability

Table S1: Country-level variables on selected aspects of cultural diversity

Table S2: Cross-country data used in analyses.

Figure S1: Pairwise correlation between country-level variables

Figure S2: Examples of variables correlated with beard frequency.

Figure S3: Examples of variables correlated with belief in the devil and parasite load

Figure S4: Global distribution of languages with a single semantic category for hand and finger

Detail of analyses described in the main text:

1. Beards and parasites

The frequency and style of male facial hair varies over time (e.g. Robinson 1976) and space (e.g. Dixson et al. 2017). A number of evolutionary hypotheses have been put forward for variation in beard frequency between cultural groups, particularly concerning intersexual selection (women's preferences for bearded men: e.g. Marcinkowska et al. 2019) and intrasexual selection (beards as a marker of likely success in male-male competition: e.g. Dixson et al. 2018). Drawing on the Hamilton-Zuk theory of costly male ornaments, it has been suggested that beards may function, like colourful tail feathers or mating displays, as markers of relative health. This hypothesis has been supported by showing that, for a sample of 25 countries, beard frequency increases with increasing parasite load and also higher income inequality (Dixson and Lee 2020; Pazhoohi and Kingstone 2020). Regardless of whether the proposed cultural evolutionary explanations of variation in beard frequency are true or not, these correlations do not provide particularly convincing evidence of a causal connection, because significant correlations with beardiness are not difficult to generate, even with variables that do not seem to have any convincing causal connection with cultural selection favouring men with facial hair. Neighbouring cultures share many factors in common, including environmental factors (like parasite load), norms of appearance (like beards), socioeconomic factors (like income inequality), spiritual beliefs (like belief in the devil) and many other traits. Any trait that tends to be similar between neighbours could be significantly correlated with any other trait that is similar between neighbours.

We added a number of cultural and socioeconomic variables to the data from Pazhoohi & Kingstone (2020) in order to identify significant associations with beard frequency, parasite load and income inequality. These variables were chosen to represent cultural factors that are likely to show non-random patterns in space but no direct causal connection to beards or parasites, for example number of Olympic medals or cheese consumption patterns. Each variable and the source from which data were obtained are described in Table S1, and the data are presented in Table S2. To identify variables that correlate with beard prevalence and parasite load, we computed a matrix of Spearman's ρ rank correlations for all possible pairs of variables and selected a sample of those which showed a significant correlation to plot in Figure S2. We used Spearman's ρ because, as a non-parametric test, we were able to apply the same

analysis to all pairs of variables regardless of whether one or more of those variables were non-normally distributed. Pairwise correlations are given in Figure S1.

Beard frequency is significantly correlated with historical disease prevalence (Spearman's rank correlation test; N=22, ρ = 0.479, p =0.02), but also with alcohol consumption per capita (N=22, ρ = -0.429, p=0.05) and with the number of nurses and midwives per 1000 people (N=22, ρ = -0.815, p<0.001). In fact, current population figures provide as strong a correlation with proportion of men with beards as parasite stress (N=22, ρ=0.486, p=0.02) (Figure S2). Similarly, beard frequency has been linked to income inequality, as a potential indicator of the degree of competition between males for mates (Dixson and Lee 2020; Pazhoohi and Kingstone 2020). But the index used to represent inequality (GINI) correlates with historical disease prevalence (N=60, ρ=0.391, p=0.002), so, unsurprisingly, GINI also correlates with many of the same variables as both beards and parasites, such as number of nurses and midwives (N=59, ρ=-0.335, p=0.01) and population size (N=60, ρ=0.408, p= 0.001). Since beard frequency, population size and GINI all correlate together (Figure S1), they may also correlate with anything else that correlates with any of these variables (Figure S2), such as parasite stress, climatic variables and socioeconomic factors (Bromham et al. 2018; Guernier et al. 2004; Kummu and Varis 2011).

2. Belief in the devil and parasites

A recent cross-cultural study demonstrated that pathogen prevalence is significantly correlated with beliefs about spiritual forces of evil (Bastian et al. 2019). The study reports a significant correlation between belief in the devil and historical pathogen prevalence (Figure S1 in Bastian et al 2019). The same country-level data shows just as strong support for a correlation between belief in the devil and number of traffic-related deaths per 100,000 people (N=43, ρ = 0.557, p <0.001) as it does for a correlation between belief in the devil and historical disease prevalence (N=45, ρ =0.485, p<0.001: Figure S3). Similarly, historical disease prevalence is just as strongly correlated with cheese consumption (N=30, ρ =-0.711, p<0.001) or the number of medals in the last summer Olympics (N=58, ρ= -0.43, p=0.001) as it is with belief in the devil. If statistical evidence from cross-cultural correlations in these data is being used to support a causal link between parasite stress and belief in the devil, then we have to conclude that there is just as strong evidence for a causal link between parasite stress, cheese and Olympic medals.

3. Hand/finger distinction and language endangerment

We used the 'Finger and Hand' feature (130A) from the World Atlas Linguistic Structures (WALS) online database (Dryer and Haspelmath 2013) which identifies 521 languages with different words to distinguish between 'hand' and 'finger(s)' and 72 languages which use a single word for both 'hand' and 'finger(s)' (Brown 2013). The database also includes geographic coordinates for each of the languages (Figure S4). We obtained Agglomerated Endangerment Status (AES) scores for 436 of the languages in the Finger and Hand database from the supplementary data of Bromham et al.'s (2022) study of global predictors of language endangerment. The AES includes six ordinal levels of endangerment; *not endangered*, *threatened*, *shifting*, *moribund*, *nearly extinct*, and *extinct* (Bromham et al. 2022). To test whether languages with a single word for both 'hand' and 'finger(s)' were more likely to be endangered we fitted a linear model with the AES score as a numeric outcome variable and a single binary predictor variable denoting whether a language has a single lexical category for 'hand' and 'finger(s)'. We found that languages with separate categories for hand and finger were significantly less likely to be endangered than languages with a single lexical category for hand/finger (Linear regression of endangerment level as the outcome and identical/different hand-finger categories as the predictor: N = 436, ß= -0.52, 95%CI [-0.93–-0.11], z = -2.467, p = 0.014, df = 434). To investigate whether this significant association could be a consequence of spatial autocorrelation, we refit this model with a structured variance-covariance matrix as a random effect with an exponential decay process using the *glmmTMB* R package (Brooks et al. 2017). Under this spatially explicit model, having a single lexical category for hand/finger was not significant associated with language endangerment (N = 436, ß= 0.409, 95%CI [-0.044–0.861], z = 1.771, p = 0.08, df = 431). Note that this analysis does not account for patterns of relatedness: if having a single category for hand/finger is non-randomly distributed among language families (as suggested by its pattern of geographic distribution), and if families have different proportions of endangered languages, then phylogenetic non-independence could also cause endangerment to be correlated with having a single hand/finger category.

4. Tonal languages and humidity

To test if tonal languages are more likely to occur in humid areas, we used the WALS database to derive information on tone (13A: Maddieson 2013), one of the databases used by Everett et al. (2016), which includes information on tonality for 527 languages. We simplified the tonality categories in the WALS (no tones, simple tone system, complex tone system) by treating tonality as a binary variable, i.e. a language contains some tonal elements (simple and complex) or it contains no tonal elements (no tones). For each language, we estimated mean humidity estimates using the same dataset as Everett et al. (2016), which includes humidity averages for 18,048 regularly spaced geographic points across the earth from 1949-2013 (Everett et al. 2016; Kalnay et al. 2022). To do this we took the overall average humidity score for each of these geographic points and, using the inverse distance weighted interpolation procedure as implemented by the 'interpolate' function from the R package *raster* (Hijmans 2023). This created a one-degree longitude by one degree latitude raster of global mean humidity scores. We then extracted the expected mean humidity score at the coordinates associated with each of the languages recorded in the WALS tonality database and scaled and centred these values.

Using logistic regression, we found a significant positive association between tonality and mean humidity (N = 527, ß= 0.301, 95%CI [0.123–0.483], z = 3.29, p = 0.001, df = 525) with an odds ratio of 1.351, 95%CI [1.131–1.62], i.e., an increase of one standard deviation of mean humidity means that the odds of a language being tonal increased by a factor of 1.351. A Moran's I test for distance-based autocorrelation on scaled residuals simulated from our model using the 'simulateResiduals' and 'testSpatialAutocorrelation' functions from the R package *_DHARM* (Hartig 2022) and confirmed significant spatial autocorrelation (N = 527, observed = 0.152, expected = -0.002, s.d. = 0.007, p < 0.001). To test if the association between tonality and humidity could be a consequence of these two variables showing similar spatial structuring, we fitted generalised linear mixed models with tonality as outcome variable, mean humidity as a predictor variable and a structured variance-covariance matrix as a random effect with an exponential decay process to correct for the effect of spatial autocorrelation and an underlying binomial probability distribution (Brooks et al. 2017). Under this model, humidity was not a significant predictor of tonality (N = 527, ß= 0.273, 95%CI[-0.639–1.184], OR = 1.313, 95%CI

[0.528–3.268]), z = 0.586, p = 0.558, df = 523, residual deviance = 465.4) suggesting that much of the covariation between the two variables can be explained by spatial autocorrelation.

To test whether other language features might also vary with humidity, we obtained two additional datasets from WALS. Past tense (Feature 66A: Dahl and Velupillai 2013) identifies 134 languages with some grammatical feature for distinguishing between the past and present tense and 88 languages with no such features. Passive construction (Features 107A: Siewierska 2013) identifies 162 languages with passive constructions and 211 languages without passive constructions. For both feature datasets, we extracted estimated mean humidity scores for all observed languages from the raster produced for the tonality analysis and scaled and centred these values. We then ran logistic regressions with the language feature as the outcome variable and the mean humidity score as the predictor. We found that mean humidity score was significantly negatively correlation with the presence of the past tense (N = 222, ß= -0.341, 95%CI [-0.628–-0.065], OR = 0.711, 95%CI [0.533–0.937], z = -2.385, p = 0.017, df = 220, residual deviance = 292.26), but significantly positively correlated with passive constructions (N = 373, ß= 0.668, 95%CI [0.449–0.895], OR = 1.949, 95%CI [1.567–2.446], z = 5.88, p < 0.001, df = 371, residual deviance = 472.92).

To illustrate the problem of covariation between environmental and cultural variables, we tested whether there was a correlation between a language including tonal elements and the richness of amphibian species in the area in which a language is spoken. To estimate amphibian species richness, we downloaded polygons for all available amphibian species' ranges (IUCN 2022). We then created a raster for estimated amphibian species richness using the 'raster' function in the R package *raster* (Hijmans 2023). Specifically, we divided the world into a one-degree longitude by one-degree latitude grid and counted the number of species whose ranges overlapped with each grid cell. We then extracted amphibian species richness estimates for all the coordinates recorded against each of the languages in the WALS tonality database which fell within the raster and scaled and centred these values. Logistic regression with tonality as the outcome variable and amphibian species richness as the predictor suggests a significant positive association between the two (N = 451, ß= 0.293, 95%CI [0.102–-0.494], z = 2.939, p = 0.00., df = 449, residual deviance = 615.94), i.e. a one standard deviation increase in

amphibian species richness (sd = 20.994) in the region in which a language is spoken increases the odds of that language being tonal by factor of 1.34 (95%CI [1.107–1.641]).

While we base our analysis on Everett et al. (2016), we were unable to exactly replicate their analysis due to unavailability of data (e.g. ANU Phonotactics Database is not currently available online), lack of detail on methods of data extraction (e.g. how point measures of humidity were associated with languages), and lack of detail on the analysis (e.g. analysis code is not provided). However, our results are consistent with theirs, with a significant relationship between tone and humidity when spatial distribution is not accounted for, suggesting that any differences in data or analysis structure are unlikely to explain the lack of correlation once spatial distance between observations is taken into account.

Code availability: code for repeating these analyses is available at https://github.com/keaghanjames/relatives_and_neighbours/tree/main

References cited in Supplementary Information

Bastian B, Vauclair C-M, Loughnan S, Bain P, Ashokkumar A, Becker M, Bilewicz M, Collier-Baker E, Crespo C, Eastwick PW (2019) Explaining illness with evil: pathogen prevalence fosters moral vitalism. Proceedings of the Royal Society B 286(1914):20191576

Bromham L, Dinnage R, Skirgård H, Ritchie A, Cardillo M, Meakins F, Greenhill S, Hua X (2022) Global predictors of language endangerment and the future of linguistic diversity. Nature ecology & evolution 6(2):163-173

Bromham L, Hua X, Cardillo M, Schneemann H, Greenhill SJ (2018) Parasites and politics: why cross-cultural studies must control for relatedness, proximity and covariation. Royal Society open science 5(8):181100

Brooks ME, Kristensen K, Van Benthem KJ, Magnusson A, Berg CW, Nielsen A, Skaug HJ, Machler M, Bolker BM (2017) glmmTMB balances speed and flexibility among packages for zero-inflated generalized linear mixed modeling. The R journal 9(2):378-400

Brown CH (2013) Finger and Hand. In: Dryer MS, Haspelmath M (eds) WALS Online (v2020.3). https://doi.org/10.5281/zenodo.7385533 Zenodo

Dahl O, Velupillai V (2013) The Past Tense. In: Dryer MS, Haspelmath M (eds) WALS Online (v2020.3). https://doi.org/10.5281/zenodo.7385533, Zenodo

Dixson BJ, Lee AJ (2020) Cross-cultural variation in men’s beardedness. Adaptive Human Behavior and Physiology 6:490-500

Dixson BJW, Rantala MJ, Melo EF, Brooks RC (2017) Beards and the big city: displays of masculinity may be amplified under crowded conditions. Evolution and Human Behavior 38(2):259-264

Dixson BJW, Sherlock JM, Cornwell WK, Kasumovic MM (2018) Contest competition and men's facial hair: beards may not provide advantages in combat. Evolution and Human Behavior 39(2):147-153

Dryer MS, Haspelmath M (2013) WALS Online (v2020.3) https://doi.org/10.5281/zenodo.7385533 In,

Everett C, Blasí DE, Roberts SG (2016) Language evolution and climate: the case of desiccation and tone. Journal of Language Evolution 1(1):33-46

Guernier V, Hochberg ME, Guégan J-F (2004) Ecology drives the worldwide distribution of human diseases. PLoS biology 2(6):e141

Hammarström H, Forkel R, Haspelmath M, Bank S (2022) Glottolog 4.7. Max Planck Institute for Evolutionary Anthropology. , Leipzig

Hartig F (2022) _DHARMa: Residual Diagnostics for Hierarchical (Multi-Level / Mixed) Regression Models_. R package version 0.4.6, <https://CRAN.R-project.org/package=DHARMa>.

Hijmans R (2023) Hijmans R (2023). _raster: Geographic Data Analysis and Modeling_. R package version 3.6-20, <https://CRAN.R-project.org/package=raster>.

IUCN (2022) The IUCN Red List of Threatened Species. Version 2022-2. https://www.iucnredlist.org. Accessed on [03 March 2023]. In: Nature IUftCo (ed),

Kalnay E, Kanamitsu M, Kistler R, Collins W, Deaven D, Gandin L, Iredell M, Saha S, White G, Woollen J, Zhu Y, Leetmaa A, Reynolds B, Chelliah N, Ebisuzaki W, Higgins W, Janowiak J, Mo KC, Ropelewski C, Wang J, Jenne R, Joseph D (2022) Dataset: NOAA NCEP-NCAR CDAS-1 MONTHLY Diagnostic Above_ground.” http://iridl.ldeo.columbia.edu/SOURCES/.NOAA/.NCEP-NCAR/.CDAS-1/.MONTHLY/.Diagnostic/.above_ground/ In,

Kummu M, Varis O (2011) The world by latitudes: A global analysis of human population, development level and environment across the north–south axis over the past half century. Applied geography 31(2):495-507

Maddieson I (2013) Tone. In: Dryer MS, Haspelmath M (eds) WALS Online (v2020.3) https://doi.org/10.5281/zenodo.7385533

(Available online at http://wals.info/chapter/13, Accessed on 2023-03-21.). Zenodo,

Marcinkowska UM, Rantala MJ, Lee AJ, Kozlov MV, Aavik T, Cai H, Contreras-Garduño J, David OA, Kaminski G, Li NP, Onyishi IE, Prasai K, Pazhoohi F, Prokop P, Cardozo SLR, Sydney N, Taniguchi H, Krams I, Dixson BJW (2019) Women's preferences for men's facial masculinity are strongest under favorable ecological conditions. Scientific Reports 9(1):3387

Pazhoohi F, Kingstone A (2020) Parasite prevalence and income inequality positively predict beardedness across 25 countries. Adaptive Human Behavior and Physiology 6:185-193

Robinson DE (1976) Fashions in shaving and trimming of the beard: The men of the Illustrated London News, 1842-1972. . American Journal of Sociology 81:1133-1141

Siewierska A (2013) Passive Constructions (Available online at http://wals.info/chapter/107). In: Dryer MS, Haspelmath M (eds) WALS Online (v2020.3). https://doi.org/10.5281/zenodo.7385533 Zenodo

Wickham H (2016) ggplot2: Elegant Graphics for Data Analysis. https://ggplot2.tidyverse.org.. In: Springer-Verlag, New York

Table S1: Country-level variables on selected aspects of cultural diversity. Variable name is used in the plots (Figures S1, S2, S3) and table of values (Table S2). Description provides details of the variable, and Source where the country level information was derived from.

Variable name	Description	Source
Alcohol	Alcohol consumption per person per year	World Health Organisation (WHO) - Global status report on alcohol and health 2018
Beard_proportion	Proportion of survey respondents who report having a beard, derived from The World's Muslims' dataset, created and maintained by the Pew Research Center	Pazhoohi, F., & Kingstone, A. (2020). Parasite prevalence and income inequality positively predict beardedness across 25 countries. Adaptive Human Behavior and Physiology, 6, 185-193
Belief_in_Devil	Derived from World Values Survey (Wave 3)	Bastian, B., Vauclair, C.-M., Loughnan, S., Bain, P., Ashokkumar, A., Becker, M., Bilewicz, M., Collier-Baker, E., Crespo, C., & Eastwick, P. W. (2019). Explaining illness with evil: pathogen prevalence fosters moral vitalism. Proceedings of the Royal Society B, 286(1914), 20191576.
Nurses	Nurses and midwives per 1000	World Bank: https://data.worldbank.org/indicator/SH.MED.NUMW.P3
Population	Esimtated population size per country (2023), based on the latest United Nations Population Division estimates	Worldometer: https://www.worldometers.info/world-population/population-by-country
GDPpc	Gross Domestic Product per capita	World Bank: Most recent value from https://databank.worldbank.org/reports.aspx?source=2&series=NY.GDP.PCAP.CD&country=
Olympic_medals	2020 summer Olympics medal tally per country	Wikipedia: https://en.wikipedia.org/wiki/2020_Summer_Olympics_medal_table
Cheese	Per capita consumption of cheese worldwide in 2016, by country (in kilograms)	Statinvestor: https://statinvestor.com/data/28032/cheese-consumption-per-capita-worldwide-country-comparison/

Road_deaths	Deaths due to traffic accidents per 100000	World Health Organisation 2016 report via en.wikipedia.org/wiki/List_of_countries_by_traffic-related_death_rate
Parasite	Historical pathogen load, using the 9 item values, apart from Kazakhstan. Tajikistan and Kyrgystan for which only 7 item value was available.	Murray, D.R. and Schaller, M., 2010. Historical prevalence of infectious diseases within 230 geopolitical regions: A tool for investigating origins of culture. Journal of Cross-Cultural Psychology, 41(1), pp.99-108.
GINI	Income inequality, represented by the Gini index, using most recent estimates from CIA (based on data from different years)	CIA: https://www.cia.gov/the-world-factbook/field/gini-index-coefficient-distribution-of-family-income/country-comparison

Table S2: Cross country data used in analyses. For information on variables and datasources, see Table S1. Country names are taken from the original publications and may not reflect current political entities or preferred designations.

Country	Parasite	Belief_in _Devil	Beard_ proportion	GDPpc	Road_ deaths	Olympic_ medals	Cheese	Alcohol	Nurses &Midwives	Population	GINI
Albania	-0.25	0.38	0.019	6492.9	15.1	0		7.5	5.1	2877797	30.8
Algeria	0.47		0.315	3690.6	23.8	0		0.9	1.5	43851044	27.6
Australia	-0.25	0.47		60443.1	4.5	46	14.7	10.6	13.2	25499884	34.3
Argentina	-0.12	0.54		10636.1	13.6	3	11.6	9.8	2.6	45195774	42.3
Armenia	0.10	0.40		4966.5	18.3	4		5.5	4.4	2963243	25.2
Azerbaijan	0.33	0.48	0.071	5388.0	10.0	7		0.8	6.4	10139177	33.7
Bangladesh	0.62	0.96	0.273	2457.9	13.6	0		0.0	0.4	164689383	32.4
Belarus	-0.75	0.47		7302.3	13.7	7	12.9	11.2	11.0	9449323	24.4
Bosnia&Herzegovina	0.00		0.040	7143.3	17.7			6.4	5.7	3280819	33.0
Bulgaria	-0.35	0.25		12221.5	9.0	6		12.7	4.8	6,948,445	40.3
Chile	-0.45	0.60		16265.1	12.4	0	9.3	9.3	13.3	19116201	44.9
Columbia	0.27	0.41		6104.1	16.8	5	1.4	5.8	0.6	50882891	54.2
Croatia	-0.44	0.04		17685.3	7.3	8	13	8.9	6.2	4105267	28.9
Czech Republic	-0.87	0.14		26821.2	4.2	11	17.6	14.4	8.4	10708981	25.3
Egypt	0.44		0.161	3698.8	12.8	6	4.2	0.4	1.9	102334404	31.5
El Salvador	0.29	0.74		455.1	21.1	0		3.7	1.8	6486205	38.8
Estonia	-0.62	0.26		27943.7	7.0	2	20	11.6	6.6	1326535	30.8
Georgia	0.10	0.60		5023.3	11.8	8		9.8	5.2	3989167	34.5
Germany	-0.87	0.17		51203.6	3.7	37	24.7	13.4	13.5	83783942	31.7

Finland	-0.75	0.48		53654.8	3.8	2	27.3	10.7	14.9	5540720	27.7
Hungary	-1.00	0.25		18728.1	6.2	20	13.2	11.4	5.3	9660351	30.0
India	0.94	0.39		2256.6	16.6	7		5.7	2.4	1380004385	35.7
Indonesia	0.63		0.100	4332.7	15.3	5		0.8	3.8	273523615	37.9
Iran	-0.15		0.332	4091.2	20.5	7	4.7	1.0	2.1	83992949	40.9
Japan	0.43	0.17		39312.7	4.1	58	2.4	8.0	12.7	126476461	32.9
Jordan	0.16		0.184	4103.3	7.7	2		0.7	3.3	10203134	33.7
Kazakhstan	-0.38		0.028	10373.8	24.2	8	2.4	7.7	7.3	18776707	27.8
Kosovo			0.028	5269.8		2					29.0
Kygyzstan	-0.38		0.071	1276.7	22.0	3		6.2	5.6	6524195	29.0
Latvia	-0.40	0.47		21148.2	6.9	2	19.8	12.9	4.6	1886198	34.5
Lebanon	0.36		0.253	4136.1	22.6			1.5	1.7	6825445	31.8
Lithuania	-0.75	0.58		23723.3	6.6	1	17.4	15.0	9.4	2722289	35.3
Malaysia	0.50		0.302	11109.3	23.6	2		0.9	3.5	32365999	41.1
Mexico	0.28	0.58		10045.7	12.3	4	3.9	6.5	2.4	128932753	45.4
Morocco	0.59		0.283	3975.4	18.0	1		0.6	1.4	36910560	39.5
New Zealand	-0.98	0.39		48781.0	7.8	20	8.2	10.7	11.1	4822233	36.2
Niger	0.51		0.514	590.6	26.4	0		0.5	0.2	24206644	37.3
Nigeria	1.15	0.96		2065.7	20.5	2		13.4	1.5	206139589	35.1
Norway	-0.85	0.28		89154.3	2.0	8	19.8	7.5	18.3	5421241	27.7
Pakistan	0.02	1.00	0.313	1505.0	14.2	0		0.3	0.5	220892340	29.6
Palestine			0.313								
Peru	0.23	0.69		6621.6	13.9	0		6.3	3.0	32971854	43.8
Phillipines	0.50	0.92		3460.5	10.5	4		6.6	5.4	109581078	42.3
Puerto Rico	0.07	0.79		32640.7		1				2860853	

Romania	-0.18	0.71		14848.2	9.6	4		12.6	6.1	19237691	34.8
Russia	-0.39	0.40	0.203	12194.8	11.6	71	5.7	11.7	4.5	145934462	36.0
Serbia	-0.23	0.20		9230.2	7.6	9		11.1	6.1	8737371	36.2
Slovakia	-1.00	0.41		21391.9	4.5	4	14	11.5	6.0	5459642	25.2
Slovenia	-0.87	0.27		29291.4	4.9	5		12.6	10.2	2078938	24.2
Spain	-0.05	0.41		30103.5	3.7	17	9	10.0	6.1	46754778	34.7
South Africa	0.11	0.78		7005.0	25.1	3	1.9	9.3	1.3	59308690	63.0
Sweden	-0.98	0.14		61028.7	2.2	9	20.5	9.2	12.6	10099265	28.8
Switzerland	-1.08	0.32		91991.6	2.2	13	22.2	11.5	17.9	8654622	32.7
Taiwan	0.30	0.68			12.1					23816775	33.6
Tajikistan	0.02		0.143	897.0	18.8	0		3.3	4.8	9537645	34.0
Thailand	0.64		0.320	7066.2	32.7	2		8.3	3.2	69799978	36.4
Tunisia	0.81		0.165	3807.1	24.4	2		1.9	2.5	11818619	32.8
Turkey	0.16	0.84	0.216	9661.2	12.3	13	7.8	2.0	3.0	84339067	41.9
Ukraine	-0.40	0.45		4835.6	13.7	19	3.6	8.6	6.7	43733402	26.1
Uruguay	0.39	0.27		17313.2	13.4	0	8.7	10.8	7.2	3473730	39.7
USA	-0.89	0.76		70248.6	12.4	113	16.7	9.8	15.7	331002651	41.1
Uzbekistan	-0.44		0.039	1983.1	11.5	5		2.7	11.3	33469203	36.8
Venezuela	0.48	0.58		15975.7		4		5.6	2.1	28435940	39.0

Figure S1. Spearman's rho (ρ) for pairwise correlation between country-level variables (see Tables S1 and S2). Colour of the circle indicates the direction and strength of the correlation, with blue meaning a negative ρ and red a positive ρ. Small white circles indicate that the correlation between these two variables was not significant (p-value > 0.05).

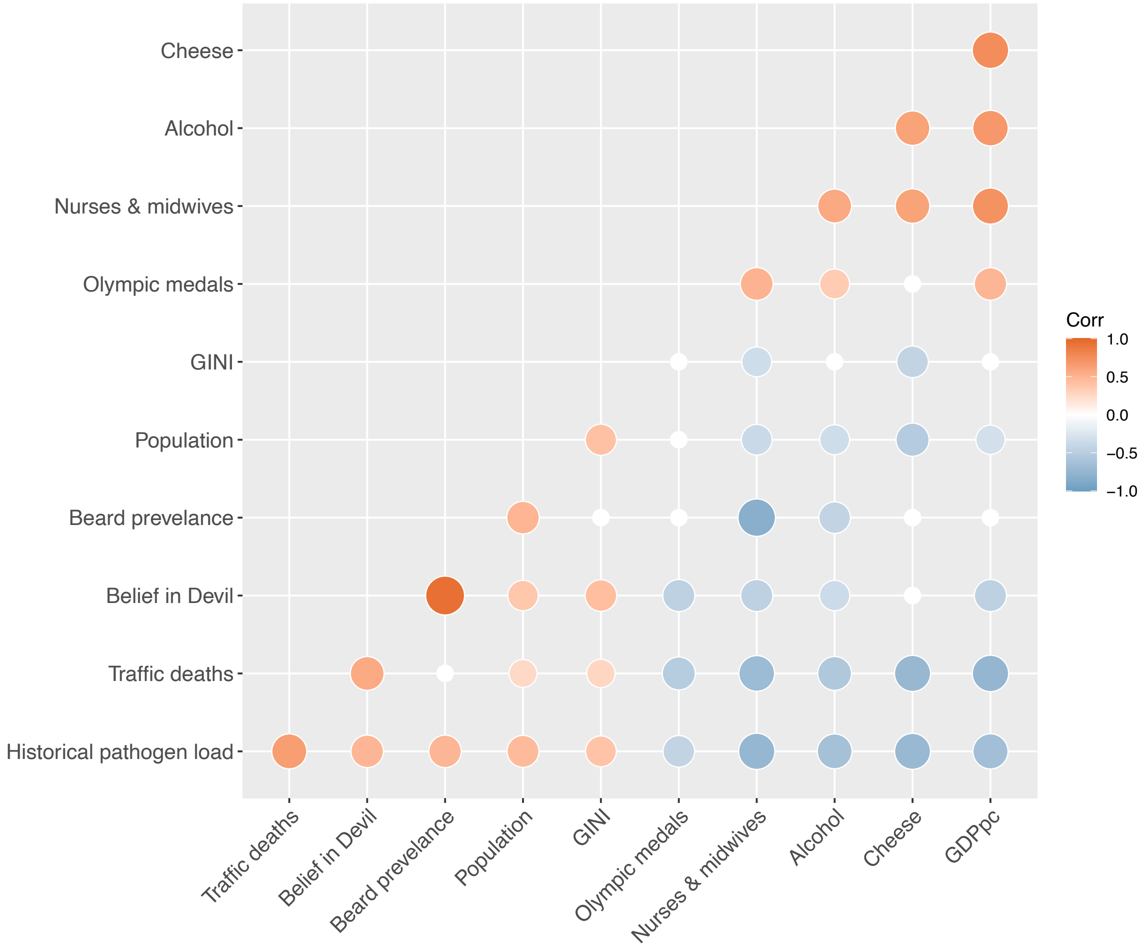

Figure S2. Examples of variables correlated with beard frequency. Prevalence of beards from survey data for 25 countries (see Pazhoohi & Kingstone, 2020) plotted against (a) historical disease prevalence; (b) alcohol consumption per capita per year; (c) nurses and midwives per thousand people; and (d) most recent population size estimates. Blue lines correspond correlation and were produced using the bivariate regression coefficient as a smoothing function (method = 'lm') as implemented in the R package 'ggplot2' (Wickham 2016).

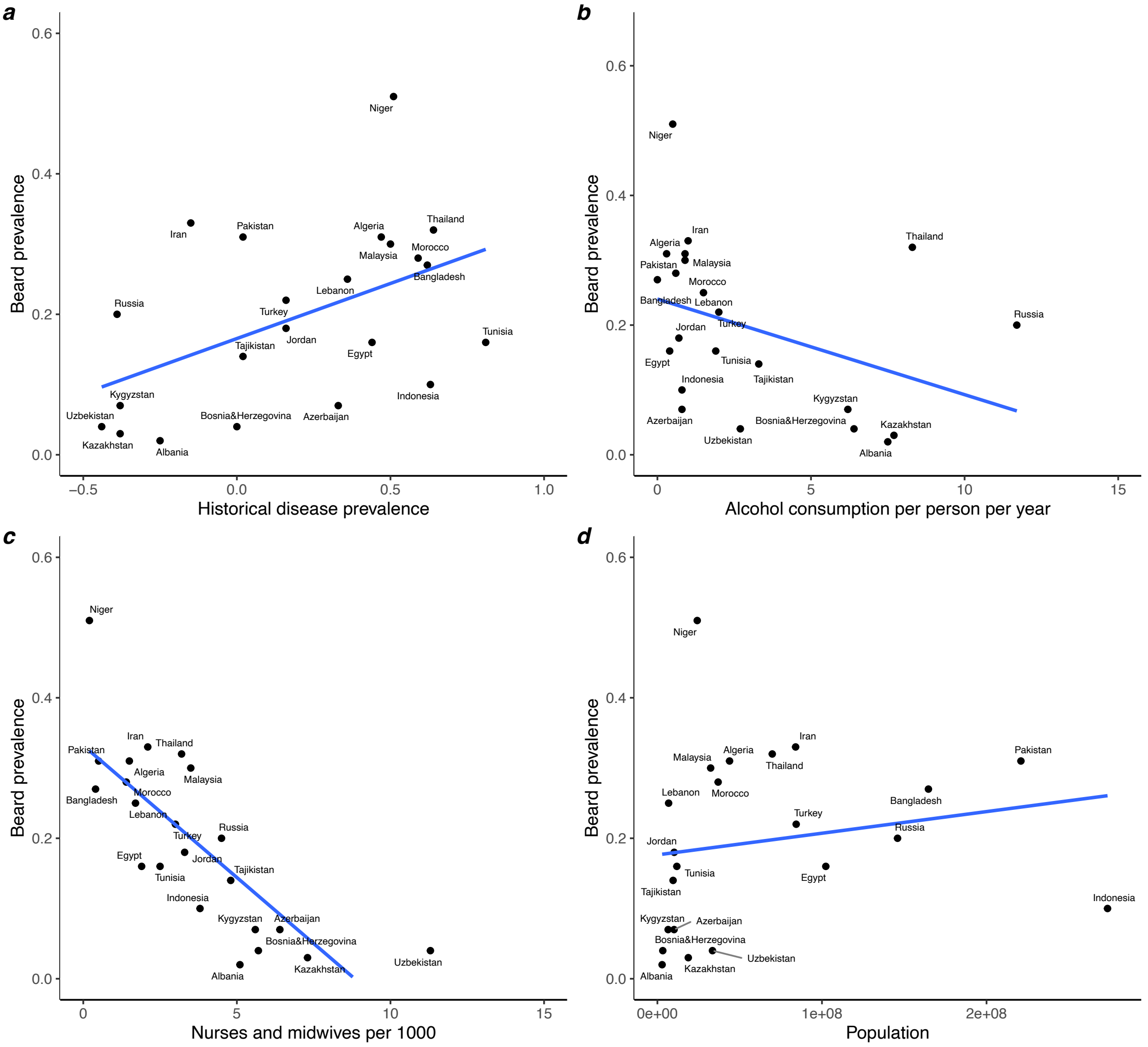

Figure S3. Examples of variables correlated with belief in the devil and parasite load. Belief in the Devil for 45 countries from Bastian et al. (2019) versus (a) historical disease prevalence and (b) deaths due to traffic accidents per 10,000 people; and historical disease prevalence against (c) cheese consumption per person per year (kg) and (d) the natural log of the number of Olympic medals in the 2020 Summer Olympics. Note that because several of these countries won no medals in the 2020 Summer Olympics, one was added to all medal tallies to aid in the visualisation of the log transformation. Blue lines correspond correlation and were produced using the bivariate regression coefficient as a smoothing function (method = 'lm') as implemented in the R package 'ggplot2' (Wickham 2016).

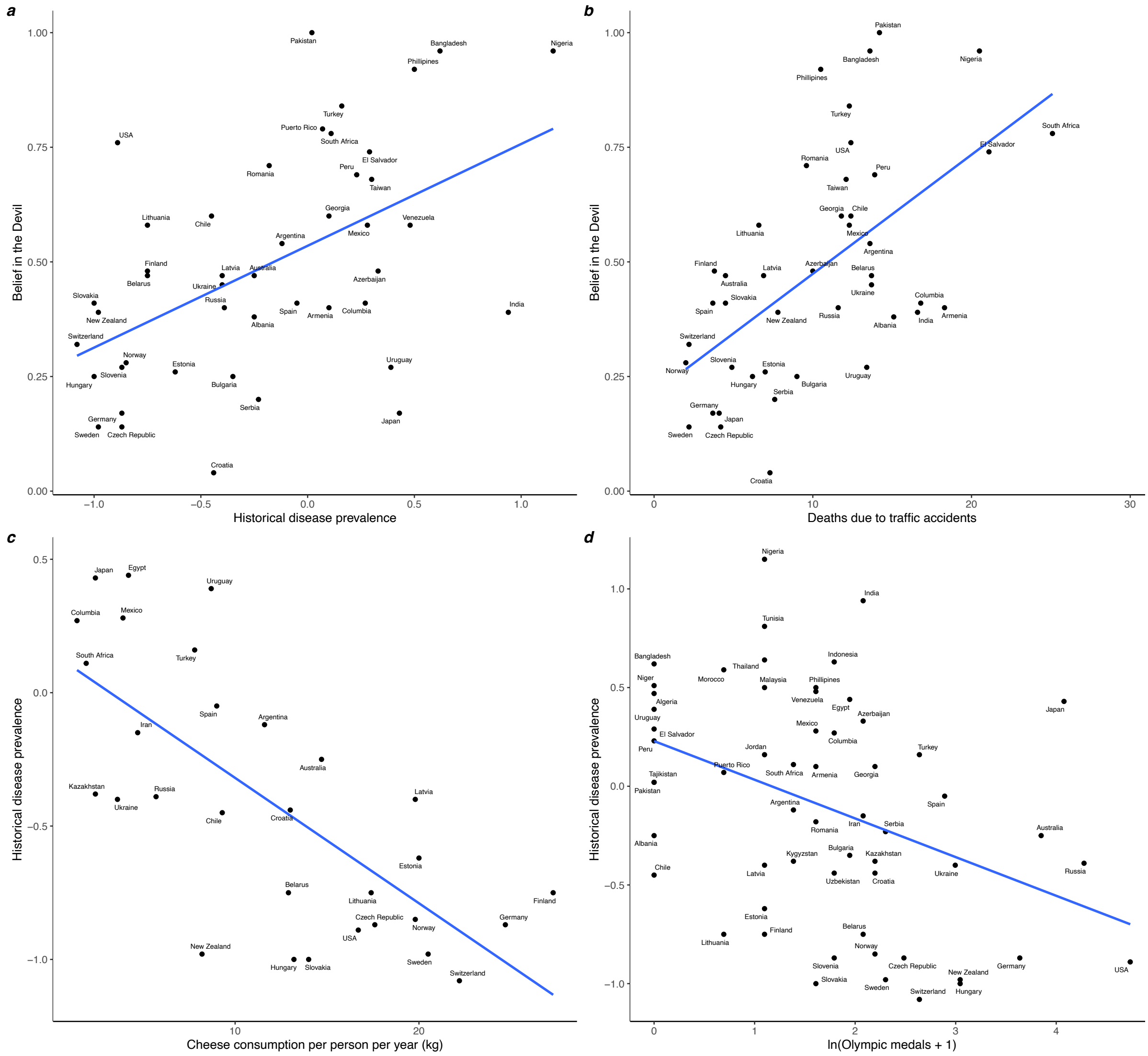

Figure S4. Global distribution of languages with a single semantic category for hand and finger (triangles) and languages with separate sematic categories for hand and finger (cross) for the 593 languages with this variable (Feature 130A: Brown 2013) recorded in the World Atlas of Linguistic Structure (WALS) database (Dryer and Haspelmath 2013). Colour of triangles and crosses indicate the endangerment status of each language from Glottolog version 4.7 (Hammarström et al. 2022) representing six ordered levels of increasing severity using the Agglomerated Endangerment Status (AES) categories: 1="*not endangered*", 2="*threatened*", 3= "*shifting*", 4= "*moribund*", 5="*nearly extinct*", 6 = "*extinct*".

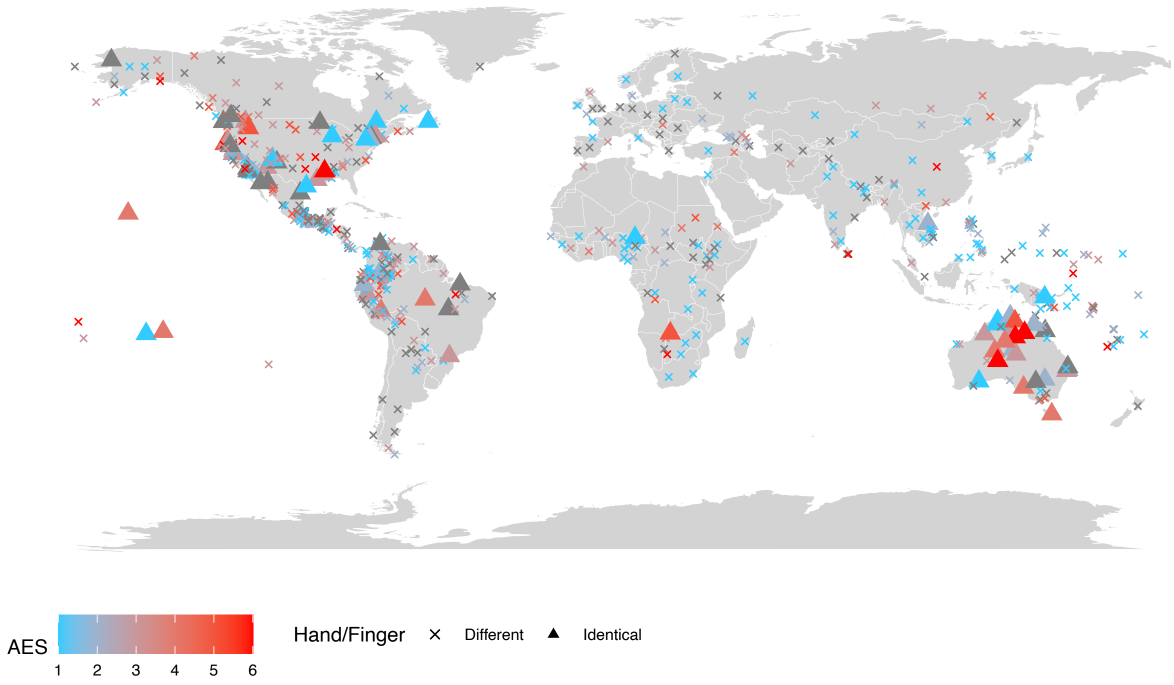

Supplement: Supplementary file 1 [file S2513843X23000233sup001.pdf]
